# A BERT-based rice enhancer identification model combined with sequence-representation differential entropy interpretation

**DOI:** 10.3389/fpls.2025.1618174

**Published:** 2025-06-09

**Authors:** Yajing Pu, Xintong Hao, Zhaoqi Zheng, Huiyan Ma, Zhibin Lv

**Affiliations:** ^1^ College of Biomedical Engineering, Sichuan University, Chengdu, China; ^2^ College of Life Sciences, Sichuan University, Chengdu, China

**Keywords:** rice enhancer, large language model, positive and negative sample distribution, support vector machine, visual explanation

## Abstract

Rice is a crucial food crop, and research into its gene expression regulation holds significant importance for molecular breeding and yield improvement. Enhancers, as key elements regulating the spatiotemporal-specific expression of genes, represent a core challenge in functional genomics due to their precise identification requirements. Current deep learning-based methods for rice enhancer identification face limitations primarily in feature extraction efficiency and the generalization capabilities of model architectures. In response, this study introduces a novel model architecture, RiceEN-BERT-SVM, which integrates DNABERT-2 as a feature extraction tool, alongside Support Vector Machine (SVM) for enhancer sequence classification. The mechanism underlying the optimization of model performance is elucidated through differential entropy analysis of feature representations. Experimental results demonstrate the high precision of this approach, achieving an accuracy of 88.05% in 5-fold cross-validation and 87.55% in independent testing. These metrics surpass current state-of-the-art (SOTA) models by margins ranging from 1.47% to 6.87% on the same dataset. Further refinement through fine-tuning enhances RiceEN-BERT-SVM's performance, increasing its accuracy by an additional 6.95%, resulting in a final accuracy of 93.63%. The study employs differential entropy analysis of sequence feature representations to explain the performance enhancements observed with increased fine-tuning iterations. As the number of iterations rises, the differential entropy distributions of positive and negative sample features gradually separate from their initial overlapping state, corresponding with the model's progressive improvement in performance. At six fine-tuning iterations, the separation between positive and negative sample entropy reaches its peak, achieving optimal model performance. Beyond this point, the distributions begin to overlap again, leading to a decline in performance. This novel approach not only offers an efficient tool for rice enhancer identification but also introduces a visually interpretable framework based on differential entropy, providing a new perspective for optimizing biological sequence analysis models.

## 1 Introduction

An enhancer is a DNA sequence in the genome that can bind to transcription factors and other regulatory proteins to enhance gene transcriptional activity (Sparks et al., 2013; Ding et al., 2023). In the rice genome, enhancers are primarily distributed in the inner regions and near gene loci, playing a crucial role in regulating gene expression (Zhao et al., 2020; Reed et al., 2023; Hamdy et al., 2024; Lin, 2024; Qiao J. et al., 2024; Zhao M. et al., 2024; Zhao Y. et al., 2024; Zhou et al., 2024). The accurate identification of rice enhancers is critical for understanding their biological mechanisms (Cao et al., 2021a; Cao et al., 2021b; Cao et al., 2022). However, traditional identification methods like chromatin immunoprecipitation sequencing (ChIP-seq) (Qiu et al., 2025) and reporter gene experiments, are labor-intensive, inefficient, and lack genome-wide coverage, making it challenging to efficiently identify enhancers throughout the genome.

In terms of computing, especially with the rise of artificial intelligence technology, machine learning-based identification of rice enhancer sequences has garnered increasing attention (Kaur et al., 2019; Machnicka and Wilczynski, 2020; Li et al., 2021; Cheng et al., 2024; Qiao B. et al., 2024; Xie et al., 2024; Yin et al., 2024). Currently, these methods can be categorized into two groups based on the distinct machine learning approaches employed. The first category comprises classic machine learning algorithms reliant on manually designed feature extraction techniques. For instance, Nisha et al. proposed the RFECS (Rajagopal et al., 2013), which integrates multi-omics features (e.g., histone modification and DNA accessibility) using a random forest model, significantly enhancing the accuracy of rice enhancer predictions. Meanwhile, Yinuo et al. introduced iEnhancer-KL (Lyu et al., 2021), combining PSTNPss and Kullback–Leibler (KL) divergence to quantify sequence distribution differences, extract nonlinear features from the rice genome, and ultimately employ SVM for enhancer classification. The second category involves deep neural networks based on automatic feature extraction (Khanal et al., 2020; Gao et al., 2022; Xiao et al., 2025). These approaches focus on improving recognition performance by leveraging various deep neural network architectures. For example, Khanal et al. developed iEnhancer-CNN (Khanal et al., 2020), which integrates word2vec models and convolutional neural networks (CNNs) from natural language processing to identify enhancers directly from raw DNA sequences (Zou et al., 2019). By contrast, Yujia et al. proposed RicENN (Gao et al., 2022), combining CNNs, bidirectional recurrent neural networks (RNNs), and attention mechanisms for the specific recognition of rice enhancers. Although these methods have achieved significant progress in enhancer recognition, they remain constrained by certain limitations. These include challenges related to model interpretability, difficulties in intuitively understanding enhancer regulatory mechanisms, inaccurate feature extraction, and limited generalization across different species.

With the innovation of the Transformer architecture, pre-trained language models have successfully expanded into the field of biomolecular sequence analysis (Liu Y. et al., 2024; Yan K. et al., 2024; Lai et al., 2025). In protein research, models such as ProtTrans (Elnaggar et al., 2022)and ESM series (Rives et al., 2021; Xiao et al., 2024) have been developed to predict protein structure and function. Similarly, in the DNA domain, several improved models based on the BERT architecture (Wei et al., 2020; Le et al., 2021; Ai et al., 2024; Li et al., 2024) exist. For example, the iEnhancer-EL proposed by Liu et al (Liu et al., 2018), employs a multi-scale k-mer labeling strategy to segment DNA sequences and extracts local semantic features of enhancer sequences using a BERT-like framework. However, the k-mer labeling method has limitations: sequence overlap leads to information redundancy, significantly increasing computational complexity, and the selection of k-values requires empirical adjustment, which limits the model’s ability to capture long-distance dependencies (Le et al., 2021; Zhou et al., 2023). In contrast, DNABERT-2, a new generation DNA language model, has achieved two major technological breakthroughs. First, it replaces traditional k-mer segmentation with the Byte Pair Encoding (BPE) word segmentation strategy. BPE dynamically merges high-frequency subsequences to generate adaptive tokens. For instance, as demonstrated in Zhihan et al.’s study, BPE encoding reduced tokenized sequence length by a factor of 5 compared to 6-mer tokenization (Zhou et al., 2023), significantly enhancing processing efficiency for long sequences. Second, its Transformer architecture incorporates Attention with Linear Biases (ALiBi) technology (Press et al., 2021), which optimizes position coding and overcomes traditional limitations on input sequence length. DNABERT-2 can flexibly process genomic sequences of any length. Therefore, when dealing with complex DNA sequences of varying lengths, DNABERT-2 demonstrates superior efficiency and accuracy in feature extraction compared to other large language models. It provides a more efficient and powerful tool for genome sequence analysis.

In information theory, information entropy measures the uncertainty and complexity of information. The larger the entropy value, the higher the disorder of the system; this corresponds to richer diversity in possible states and a greater amount of information contained (Shannon, 1948). Differential entropy extends information entropy to continuous random variables and quantifies the uncertainty inherent in their probability distributions (Kozachenko and Leonenko, 1987). If the probability density function f(x) is uniformly distributed, the differential entropy will be larger, indicating higher uncertainty. Conversely, if f(x) is highly concentrated, the entropy may be smaller or even negative. This property allows differential entropy to describe the uncertainty of continuous signals with flexibility, leading to a wide range of applications. In communication engineering, differential entropy can quantify the distribution difference between signals and noise (e.g., calculating the channel capacity limit) (Lapidoth and Moser, 2009), which is essential for optimizing efficient transmission technologies like orthogonal frequency division multiplexing (OFDM) (Li et al., 2007). In physics, differential entropy’s mathematical correspondence with thermodynamic entropy (e.g., Boltzmann’s entropy formula) provides microscopic probabilistic explanations for the analysis of macroscopic phenomena such as gas diffusion and phase transition (Ellis, 1999; Xing, 2003). In machine learning, mutual information indicators derived from differential entropy overcome the limitations of linear correlation analysis. For instance, they can capture nonlinear correlations between features and target variables. By measuring these relationships, mutual information facilitates feature screening and model performance improvement (Beirlant et al., 1997; Hanchuan et al., 2005; Leonenko et al., 2008).

To address the limitations in existing methods for rice enhancer sequence prediction regarding feature extraction and model generalization, we proposed a model called RiceEN-BERT-SVM. This model leverages DNABERT-2 as its feature extractor and employs Support Vector Machine (SVM) as the classifier. Experiments demonstrate that RiceEN-BERT-SVM efficiently identifies rice enhancers with an independent test accuracy of 87.55%, surpassing RicENN, the current state-of-the-art model, by 10.82%. By employing DNABERT-2’s fine-tuning capability for downstream tasks, we achieved a 93.63% accuracy for RiceEN-BERT-SVM after six fine-tuning iterations. We also utilize differential entropy distributions derived from positive and negative sample features to visually interpret how the performance of our fine-tuning model changes with the number of tuning iterations. Additionally, we propose a method for determining the optimal number of fine-tune iterations by analyzing the polarization of positive and negative sample-averaged differential entropy distances. Our approach not only provides novel tools and ideas for rice enhancer sequence recognition but also offers a fresh perspective on the visual interpretation of model behavior.

## 2 Materials and methods

### 2.1 Dataset

To train a rice enhancer prediction model, we obtained genome sequence data of rice (Oryza sativa Japonica Group) from the Ensembl Plants website (Quang et al., 2016; Howe et al., 2020). This database integrates DNA sequence resources from a variety of important crops, providing rich basic data for genomic research. Based on the enhancer active regions verified by Jialei et al. using STARR-seq technology (Sun et al., 2019), 9,642 enhancer sequences were extracted from rice chromosomes as positive sample. Simultaneously, based on DNase I hypersensitivity site (DHS) predictions, we identified 23,398 non-enhancer sequences as negative samples. After redundancy removal with CD-HIT tool (Huang et al., 2010; Li et al., 2012), the final dataset comprised 4,082 enhancers and 9,916 non-enhancers exhibiting a mild class imbalance with a positive-to-negative ratio of 1:2.43. The positive samples were divided into training and test sets in a ratio of 7:3. For the test set, 30% of the positive samples and an equivalent proportion of negative samples were randomly selected to maintain consistency with the original dataset’s chromosome distribution. The remaining 70% of both positive and negative samples were allocated for the training set. Ultimately, we constructed a training dataset comprising 9,346 samples and an independent test set consisting of 3,882 samples. During the training process, the training set was further subdivided into five subsets for cross-validation purposes. Of these, 80% was used to train the model, while the remaining 20% served to validate its performance.

### 2.2 Model architecture

**Figure 1:**
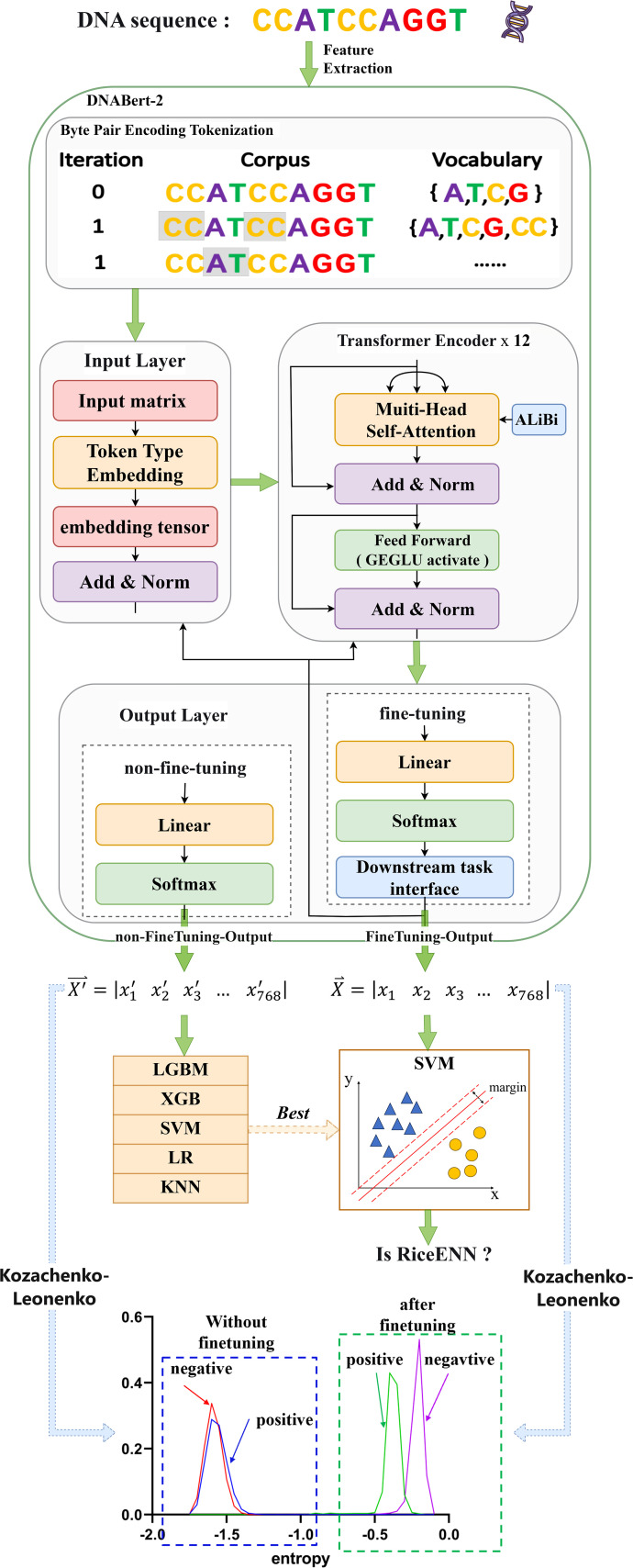
Technology roadmap. The DNA sequence was extracted by DNABert-2, and the unfine-tuned and fine-tuned feature vectors were output, respectively. After the feature sequences without fine-tuning were identified by different algorithms, it was found that the SVM algorithm had the best performance, so the features after SVM training were selected to determine whether the enhancer was not. In addition, in order to explain the effect of fine-tuning on the model, the optimal number of fine-tuning was selected, and the differential entropy of positive and negative samples was calculated and visualized.

In order to efficiently identify and predict rice enhancers, we propose a new neural network model based on DNABert-2 and explore the fine-tuned performance of the model using differential entropy, as illustrated in **Figure 1**. This framework comprises three main components: DNABert-2 feature extraction (Zhou et al., 2023), machine learning, and differential entropy computation. When entered into DNABert-2, a DNA sequence generates corresponding feature vectors for both fine-tuned and un-fine-tuned models. Subsequently, these un-fine-tuned feature vectors are inputted into five distinct machine learning algorithms. Each algorithm offers unique advantages in identifying rice enhancers. Results indicate that the SVM algorithm (Hearst et al., 1998; Wang et al., 2024) demonstrates superior performance in classifying rice enhancers. Consequently, we utilize the SVM algorithm to train using the fine-tuned eigenvectors (epoch1-epoch10), thereby obtaining predictions of rice enhancers. Additionally, considering the eigenvector without fine-tuning (epoch0) alongside the 10 epochs of fine-tuning, we estimate differential entropy employing the Kozachenko-Leonenko method (Kozachenko and Leonenko, 1987). This analysis elucidates the impact of fine-tuning through mean and median entropy differences observed in positive and negative samples before and after fine-tuning.

#### 2.2.1 Feature extraction

In DNABert-2, the input layer receives DNA sequences processed through BPE tokenization and embeds them into a high-dimensional space. These embeddings are then passed through 12 Transformer Encoder layers, which serve as the core components responsible for capturing long-distance dependencies and complex patterns within the sequences. Each Transformer Encoder layer incorporates ALiBi (Attention with Linear Biases), a positional encoding mechanism that introduces linearly decaying bias to attention weights based on token distances. This design enables flexible handling of variable-length sequences while maintaining computational efficiency. Additionally, the feedforward layers employ GEGLU (Gated Linear Unit with GELU), which splits the input into two components, applies GELU to one, and multiplies them. This gating mechanism improves nonlinear modeling compared to standard activations (Zhou et al., 2023). In the output layer, a 768-dimensional eigenvector is generated. This un-fine-tuned result from the base architecture is designated as epoch0. If subjected to further fine-tuning and optimization, these eigenvectors can serve as inputs for subsequent tasks. Within this study, the model undergoes a total of 10 training iterations following fine-tuning to yield epochs 1 through 10.

#### 2.2.2 Machine learning

**Figure 2:**
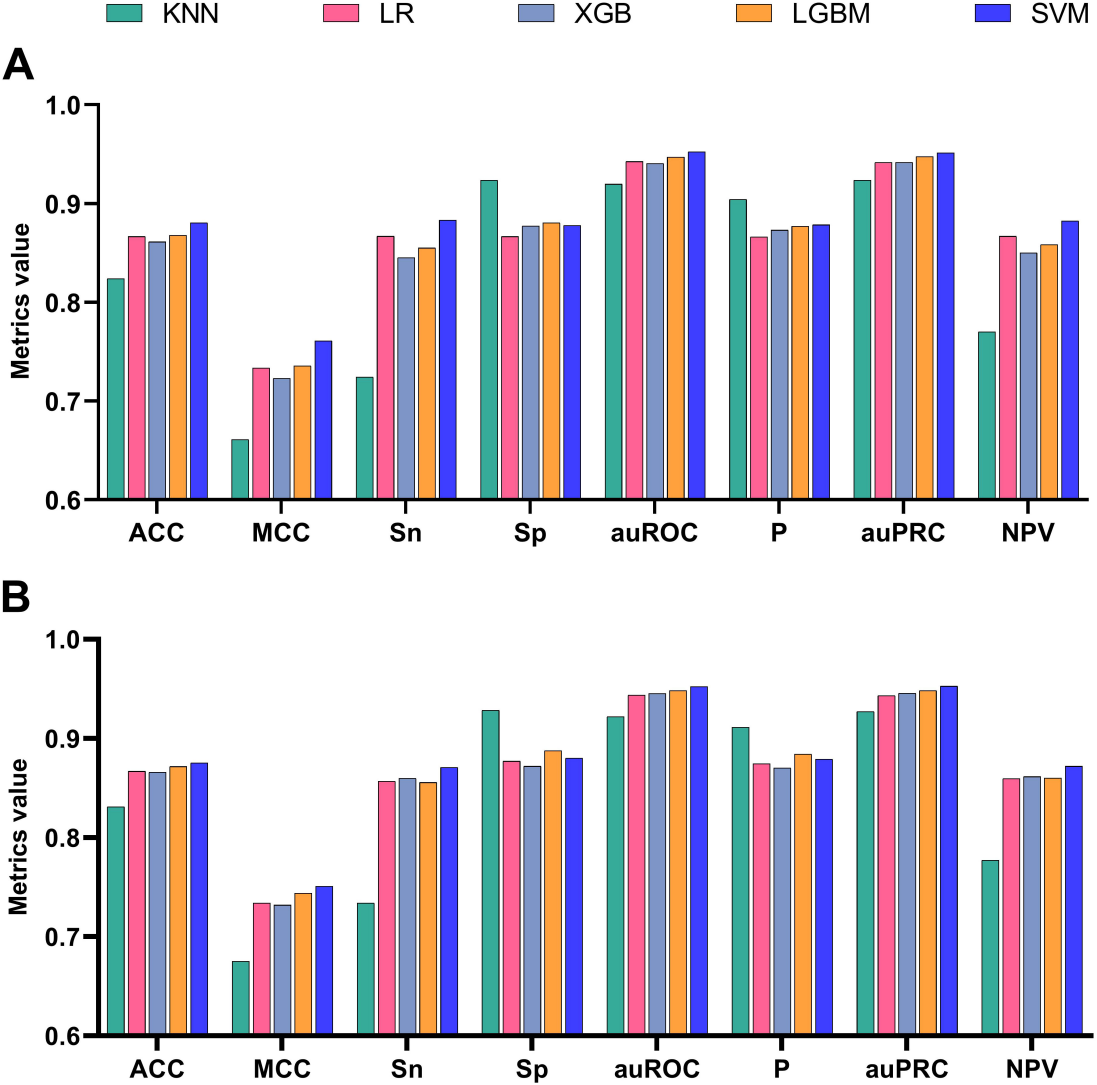
Comparison of indicators without fine-tuning for different methods: **(A)** Comparison on the training set, **(B)** Comparison on the test set.

Distinct machine learning algorithms offer various advantages in identifying rice enhancers. We evaluated five common algorithms to preprocess feature vectors derived from Bert: LGBM and XGB, both ensemble methods utilizing gradient boosting, are effective for complex nonlinear relationships, ideal for large datasets, and yield high prediction accuracy (Zou et al., 2023). SVM excels in high-dimensional spaces and small samples by finding an optimal hyperplane for classification (Zhu et al., 2023). LR is efficient and straightforward, suitable for linearly separable data, providing model interpretability. KNN classifies based on nearest-neighbor similarity, making it apt for tasks with limited data volumes. As shown in **Figure 2**, SVM outperforms other algorithms across ACC, MCC, Sn, Sp, and Sr metrics for both training and test sets. Consequently, we employed SVM to train the fine-tuned feature vectors. To ascertain the optimal fine-tuning stage for rice enhancer recognition, we input feature vectors from 10 epochs into SVM. This process yielded results for recognizing enhancers from epoch1 to epoch10, facilitating our determination of the best model performance.

#### 2.2.3 Differential entropy calculation

In information theory, differential entropy is used to measure the uncertainty of a continuous random variable, specifically, defined as follows: for a continuous random variable X with probability density function f(x) , the differential entropy h(X) (Equation 1) is defined as:


(1) $$h(X)=-\int_{-\infty }^{\infty }f(x)\text{log}f(x)dx$$

This study introduces differential entropy to quantify the ability of DNABert-2 to capture sample features during the learning process, as well as its ability to distinguish between positive and negative samples with varying numbers of fine-tuning times.

However, in practice, the probability density function (PDF) of continuous variables is typically not directly obtainable, necessitating entropy estimation from empirical data. Common approaches include binning-based discretization methods (Alneberg et al., 2014) and kernel density estimation (KDE) (Parzen, 1962). However, binning requires arbitrary discretization of data into intervals, risking information loss or artificial patterns, while KDE suffers from sensitivity to bandwidth selection and high computational costs in high-dimensional spaces. In contrast, the Kozachenko-Leonenko (K-L) entropy estimator circumvents these limitations without requiring explicit PDF estimation (Kozachenko and Leonenko, 1987; Bulinski and Dimitrov, 2019). Specifically, its core principle involves calculating the average distance from each sample point to its k-th nearest neighbor. This approach cleverly bypasses direct density modeling while demonstrating superior efficiency and accuracy in high-dimensional data processing. The formula (Equation 2) is as follows:


(2) $$H_{N}:=d\text{log}\bar{\rho }+\text{log}V_{d}+\gamma +\text{log}(N-1)$$

Where $H_{N}$ is the estimate of differential entropy H(f) . d is the dimension of the space where the random vector is located. $\bar{\rho }$ is the geometric mean of the nearest neighbor distance in the sample, that is, $\bar{\rho }={\left(\rho_{1}\cdot \rho_{2}\cdot \ldots \cdot \rho_{N}\right)}^{1/N}$ , where $\rho_{i}$ is the distance from the *i* -th sample point to its nearest neighbor sample point. $V_{d}$ is the d-dimensional unit sphere volume, that is, $V_{d}=\frac{\pi^{\frac{D}{2}}}{\text{Γ}(1+\frac{D}{2})}$ . γ is the Euler-Marshalloni constant, which is approximately equal to 0.5772. N is the sample size.

During the fine-tuning process, the differential entropy of the positive and negative samples in each epoch (from 0 to 10) is calculated using Kozachenko–Leonenko, and then the differential entropy of the positive and negative samples changes with the increase in the number of fine-tunings.

### 2.3 Model evaluation

To comprehensively evaluate the model’s performance in rice enhancer recognition, we adopted a 5-fold cross-validation approach combined with independent testing. Based on the training set and test set constructed in the previous section, during the training phase, the training set is divided into 5 subsets. Each time, 4 subsets are used for model training, and the remaining subset is used to validate the model’s performance. This process is repeated 5 times (once for each subset as the validation set) to optimize the parameters. Finally, the trained model is tested on the independent test set to obtain the prediction results.

The following metrics were selected to evaluate model performance: accuracy (ACC) (Equation 3), Matthews correlation coefficient (MCC) (Equation 4), recall (Sn) (Equation 5), specificity (Sp) (Equation 6), negative predictive value (NPV) (Equation 7), precision (P) (Equation 8), auROC, and auPRC (Grau et al., 2015; Liu M. et al., 2024; Zhu et al., 2024; Huang et al., 2025; Zhang et al., 2025). These metrics measure the classification performance of the model from different perspectives, and they are defined below:


(3) $$ACC=\frac{TP+TN}{TP+TN+FP+FN}$$

(4) $$MCC=\frac{TP\times TN-FP\times FN}{\sqrt{\left(TP+FP)(TP+FN)(TN+FP)(TN+FN\right)}}$$

(5) $$Sn=\frac{TP}{TP+FN}$$

(6) $$Sp=\frac{TN}{TN+FP}$$

(7) $$NPV=\frac{TN}{TN+FN}$$

(8) $$P=\frac{TP}{TP+FP}$$

Where TP represents true positive (TP), TN represents true negative (TN), FP represents false positive (FP), and FN represents false negative (FN). auROC stands for area under the ROC curve, which plots recall (Sn) and false positive rate (FPR) at different thresholds. Values closer to 1 indicate better performance. Similarly, auPRC stands for area under the precision-recall curve, which plots precision (P) and recall (Recall) at different thresholds; higher values closer to 1 indicate better performance.

## 3 Results and discussions

### 3.1 Analyzing ML models with pretrained LM feature extraction

The DNABERT-2 large language model employs a dynamic compression algorithm in place of the fixed k-mer window and implements the ALiBi attention mechanism to facilitate full-sequence modeling. Following extensive training on big data, it effectively captures potential feature information within sequences. Leveraging this capability, DNABERT-2 was applied to feature extraction in rice enhancer recognition tasks to evaluate its practical effectiveness in identifying rice enhancers. Simultaneously, the extracted features were entered into five distinct machine learning algorithms for classification purposes; their performances were then compared to identify the most suitable algorithm for future fine-tuning.

The experimental findings are illustrated in **Figure 2**. Figure A presents a performance comparison of the five machine learning algorithms during cross-validation, while Figure B evaluates them under independent testing conditions. It is evident that SVM emerges as the top performer across six out of eight metrics, excluding Sp and P, demonstrating consistent superiority irrespective of whether it was trained on or tested against datasets. In cross-validation, the SVM algorithm achieved a peak performance of 0.883, whereas in independent testing, its score reached 0.879. The outcomes of all eight metrics were averaged across both validation methods, reinforcing SVM’s superior performance. Compared to the other four algorithms, SVM exhibited improvements ranging from 1.41% to 6.27% during cross-validation and 0.49% to 5% during independent testing. Consequently, the SVM algorithm was identified as the most effective for the rice enhancer recognition task and selected as the foundational model for subsequent fine-tuning exercises.

### 3.2 Model fine-tuning effects

**Figure 3:**
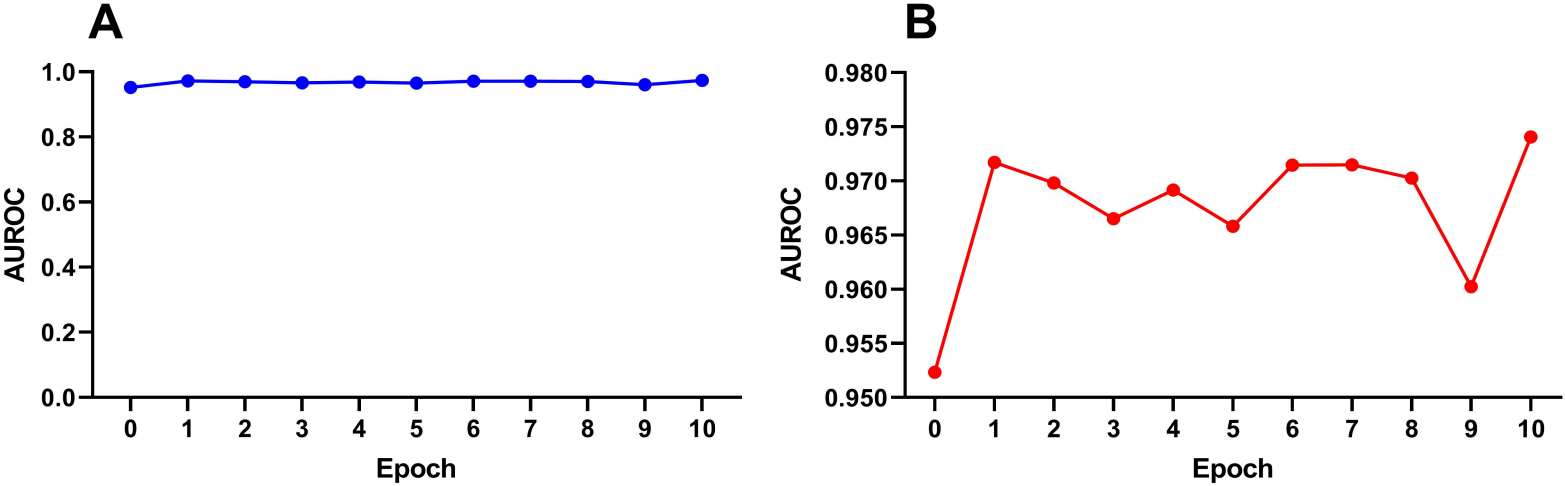
Variation trend of auROC in the testset with the number of fine-tuning times under SVM. **(A)** Full-scale view (y-axis: 0–1) demonstrating performance improvement through fine-tuning. **(B)** Magnified view (y-axis: 0.950–0.980) highlighting nuanced auROC fluctuations.

DNABert-2 can be fine-tuned for the downstream task of identifying rice enhancers. The model was fine-tuned for 10 iterations, with each fine-tuning run denoted as Epoch_i (i = 1, 2,…, 10), while the unmodified baseline was labeled Epoch_0. To mitigate overfitting risks, we prioritized evaluation on an independent test set. The performance of all 11 epochs (Epoch_0 to Epoch_10) was systematically compared, and the results are shown in **Figure 3**. Among the 8 evaluation metrics, AUC (Area Under the ROC Curve) was selected as the primary metric due to its threshold independence, which comprehensively aggregates model performance across all classification thresholds without requiring arbitrary cutoff selection. Additionally, AUC directly quantifies the model’s ranking ability, aligning with the practical need for enhancer identification in genomic studies.

**Figure 3A** demonstrates progressive performance improvement through fine-tuning at the macro scale (0–1 auROC range), while the magnified view in **Figure 3B** (0.950-0.980 auROC range) reveals performance oscillations. Our analysis suggests no clear correlation exists between model performance and the number of fine-tuning iterations, as the performance improvement does not scale linearly with additional fine-tuning epochs. During the first fine-tuning step (Epoch_1), there was a significant improvement in performance compared to Epoch_0. After that, the AUC fluctuated within a narrow range of 0.965–0.975 until Epoch 9, after which the AUC dropped significantly. It is hypothesized that excessive fine-tuning at this stage may lead to model overfitting, thereby reducing its generalization ability on the test set. At this point, we cannot definitively determine the optimal number of fine-tuning epochs.

### 3.3 Differential entropy explanation

**Figure 4:**
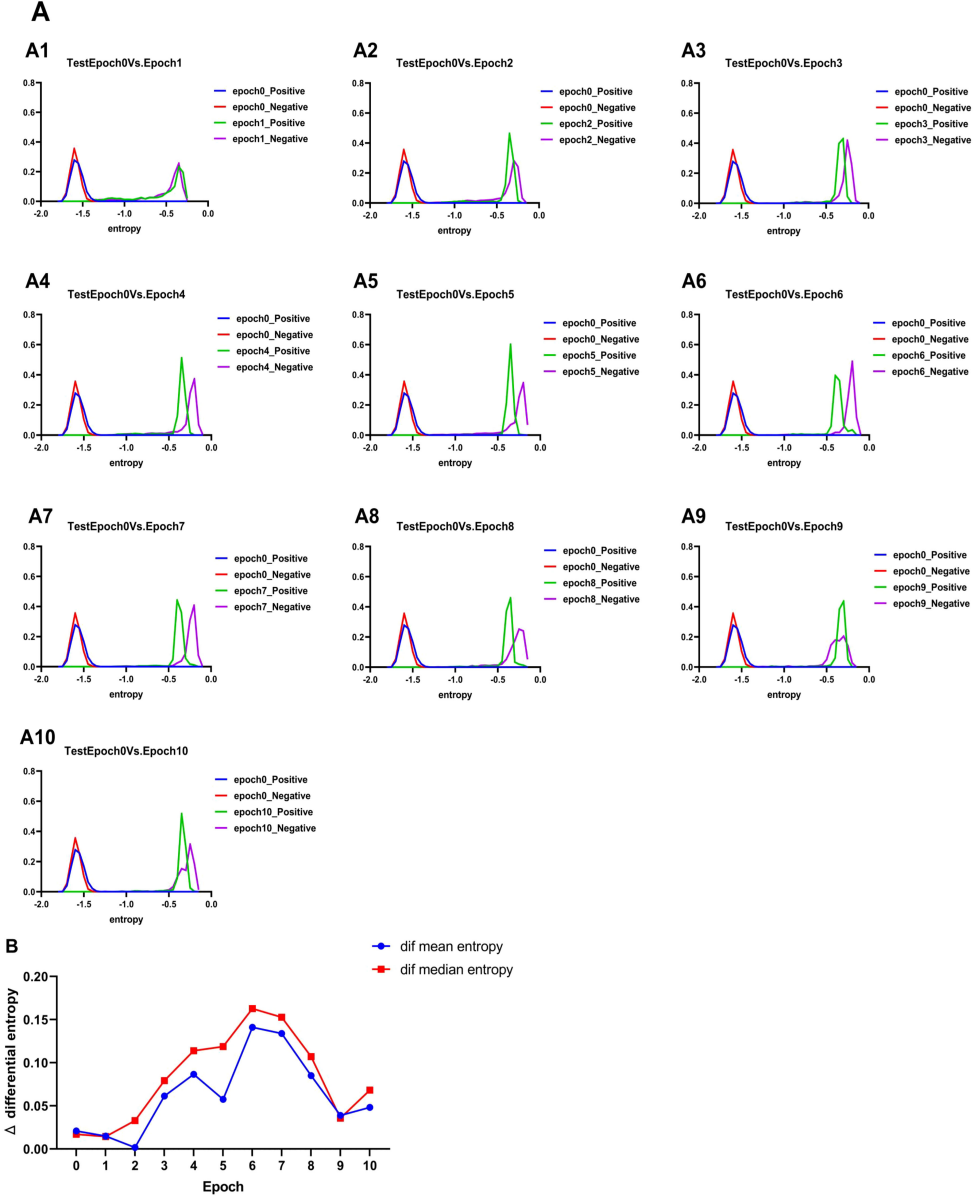
Results of differential entropy. **(A)** Change in entropy of the test set. **(B)** Change of the mean entropy difference and median entropy difference of positive and negative samples in the test set with the increase of epoches (0–10 eopoches). Where Δmean differential entropy =Positive sample mean differential entropy - Negative sample mean differential entropy |, Δmedian differential entropy =Positive sample median differential entropy - Negative sample median differential entropy |.

We calculate differential entropy to more intuitively explain the effect of fine-tuning on the model. Differential entropy measures the uncertainty of data distributions and can be used to evaluate the model’s ability to distinguish between positive and negative samples. Specifically, we used the Kozachenko–Leonenko estimator to calculate the differential entropy of the positive and negative samples for Epoch_i(i=0,1,2… 10), visualizing them to observe the change patterns. As shown in **Figure 4A**, with an increase in the number of fine-tuning epochs, the differential entropy of positive and negative samples exhibits a trend of “coincidence–separation–coincidence.” At the initial stage, the differential entropy of positive and negative samples coincided, indicating that the model had not yet fully distinguished between them. During the fine-tuning process, the differential entropy of positive and negative samples gradually separated, suggesting that the model’s ability to differentiate improved. Notably, during Epoch_6–Epoch_8, the positive and negative samples were most distinct in terms of differential entropy, which also corresponds to the “plateau” phase observed for Epoch_6–Epoch_8 in **Figure 3**. In the later stages, the differential entropy of positive and negative samples began to coincide again, likely because the model started overfitting and lost its generalization ability. At this point, it can be preliminarily determined that there is an optimal number of fine-tuning epochs between Epoch_6–Epoch_8.

To determine the optimal number of fine-tuning epochs, we calculated the mean and median differential entropy of positive and negative samples in each cycle. These values were used to compute the mean entropy difference and median entropy difference for Epoch_i, which are presented in **Figure 4B**. The results indicate that the average and median entropy differences between positive and negative samples achieve their maximum at the 6th training cycle (Epoch_6). This suggests that the model exhibits its strongest ability to distinguish between positive and negative samples during this cycle. Based on these findings, Epoch_6 was identified as the optimal model for extracting rice enhancer features.

### 3.4 Comparisons with the existing methods

**Figure 5:**
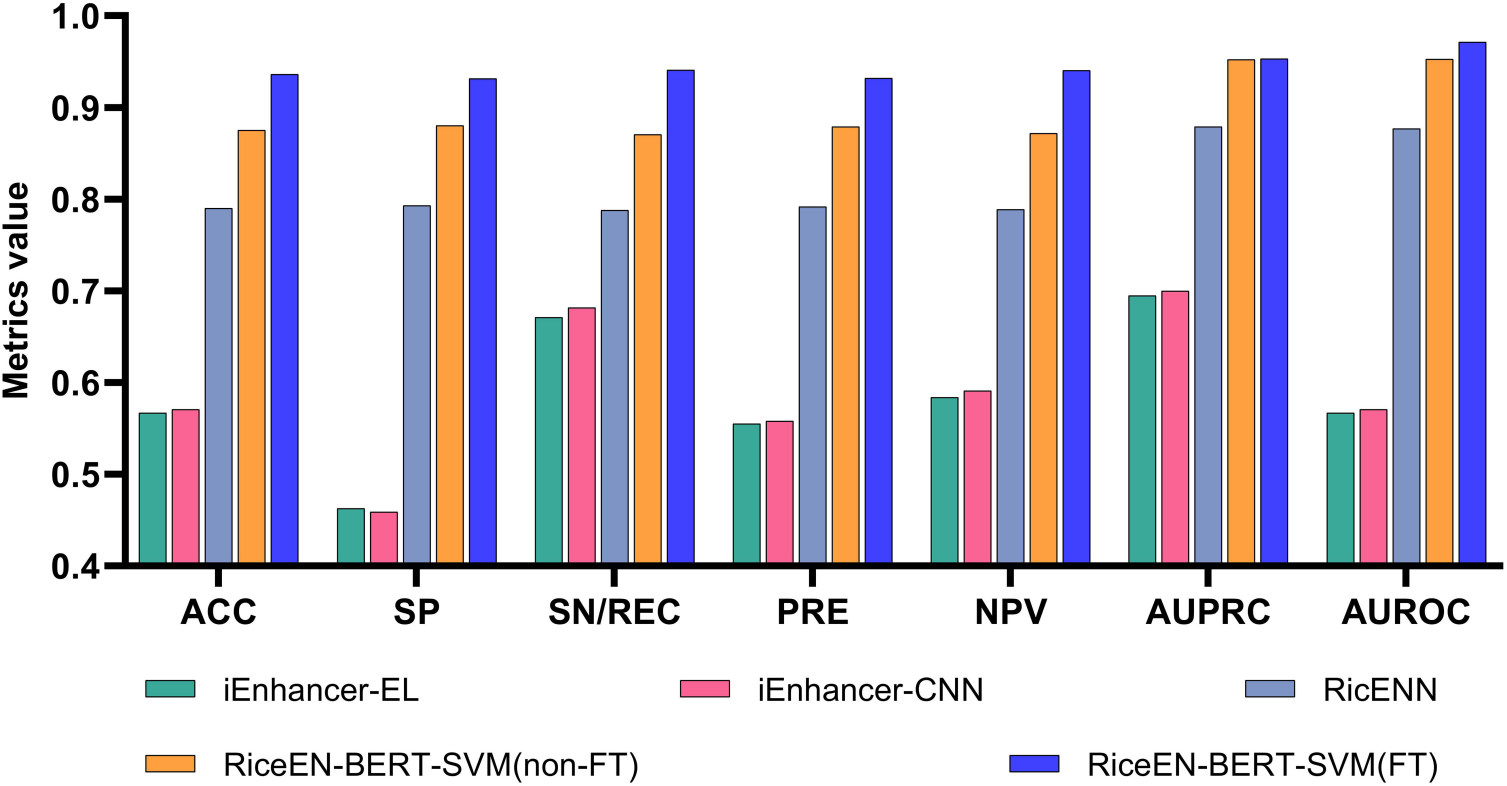
This study is compared with other methods. Our model is RiceEN-BERT-SVM, non-FT means that the model is not fine-tuned, and FT means the fine-tuned model.

Based on the best model selected above, as well as the previously unfine-tuned RiceEN-BERT-SVM, we compared our models with other existing rice enhancer recognition methods on an independent test set, including RicENN (Gao et al., 2022), iEnhancer-CNN (Khanal et al., 2020), and iEnhancer-EL (Liu et al., 2018). **Table 1** and **Figure 5** present the comparative performance metrics between our framework and state-of-the-art methods. The results demonstrate that both model configurations of RiceEN-BERT-SVM—the fine-tuned (6 iterations) and baseline (unfine-tuned) versions—consistently surpass all existing approaches across evaluation metrics. They achieved first and second places in all 7 evaluation criteria, with significant improvements in performance. Notably, the fine-tuned RiceEN-BERT-SVM demonstrated exceptional average performance during independent testing, with specific metric values as follows: ACC, SP, SN/REC PRE, NPV, AUPRC, and AUROC were 0.936, 0.932, 0.941, 0.932, 0.940, 0.953, and 0.971, respectively. Compared to RicENN, which ranked third, the performance metrics for our fine-tuned model improved by 8.42% to 19.42%. These results clearly demonstrate that the fine-tuned Bert-2 framework is effective in recognizing rice enhancers.

**Table 1:** Comparison of our methods with other methods on the independent testset.

Method	ACC	SP	SN/REC	PRE	NPV	AUPRC	AUROC
iEnhancer-EL	0.567	0.463	0.671	0.555	0.584	0.695	0.567
iEnhancer-CNN	0.571	0.459	0.682	0.558	0.591	0.700	0.571
RicENN	0.790	0.793	0.788	0.792	0.789	0.879	0.877
RiceEN-BERT-SVM(non-FT)	0.875	0.880	0.871	0.879	0.872	0.952	0.953
RiceEN-BERT-SVM(FT)	**0.936**	**0.932**	**0.941**	**0.932**	**0.940**	**0.953**	**0.971**

Bold values are the models that achieve the best performance.

## 4 Conclusion

We used DNABERT-2, a pre-trained large language model for biological sequences, to extract features from rice enhancer sequences. Combining these features with a support vector machine (SVM) classifier, we constructed a novel model, RiceEN-BERT-SVM, designed to identify rice enhancers. The model demonstrated exceptional performance, achieving cross-validation and independent test results that significantly outperformed existing state-of-the-art methods. Specifically, on the independent test set, our model achieved an accuracy (ACC) of 93.63% and an area under the receiver operating characteristic curve (AUROC) of 97.15%. These metrics represent improvements of 18.52% and 10.77%, respectively, compared to RicENN. To further understand model performance, we developed a methodology to visualize the discriminative ability of the model by leveraging the differential entropy representation of large-language-embedding features derived from DNA sequences. During fine-tuning experiments, we observed that with an increasing number of fine-tuning iterations, the differential entropy between positive and negative samples initially separated and then converged. This trend indicated that the model’s discriminative capacity first increased and later weakened as fine-tuning progressed. At a specific fine-tuning threshold (6 iterations), the difference in differential entropy between positive and negative samples was maximized, coinciding with peak model performance. Our findings demonstrate two key insights: First, pre-trained large language models like DNABERT-2 can significantly enhance the recognition of rice enhancer sequences. Second, the changes in fine-tuning performance are closely tied to shifts in the representation of positive and negative sample distributions, as captured by differential entropy. The approach of visualizing differential entropy in feature representations is broadly applicable and can serve as a valuable tool in future studies involving machine learning for DNA, RNA, or protein sequence recognition. However, our study has limitations. For instance, the differential entropy analysis does not account for spatial patterns in DNA sequences, potentially overlooking biologically meaningful structural dependencies. Additionally, the computational efficiency of the current framework can be further optimized. Future studies could develop entropy metrics integrating spatial sequence context and design lightweight architectures to enable broader genomic applications, thereby advancing efficient computational tools for crop molecular design.

## Data Availability

The raw sequence data used in the study were obtained from the following URL: https://plants.ensembl.org/index.html.
